# Marked seasonality of *Cyclospora cayetanensis* infections: ten-year observation of hospital cases, Honduras

**DOI:** 10.1186/s12879-016-1393-6

**Published:** 2016-02-04

**Authors:** Rina Girard Kaminsky, Javier Lagos, Gabriela Raudales Santos, Samuel Urrutia

**Affiliations:** 1 Pediatric Department, School of Medical Sciences, National Autonomous University of Honduras and Parasitology Service, University Hospital, Tegucigalpa, Honduras; 2 School of Medical Sciences, National Autonomous University, Honduras, Barrio Villa Adela, Casa No. 2453, Comayagüela, Honduras; 3 School of Medical Sciences, National Autonomous University, Honduras, Hospital Santa Teresa, Comayagua, Honduras; 4 School of Medical Sciences, National Autonomous University, Honduras, Barrio El Calvario, Casa No. 585, Santa Rosa de Copán, Honduras

**Keywords:** *Cyclospora*, Cyclosporiasis, Hospital care, Honduras, Seasonality

## Abstract

**Background:**

Document seasonality occurrence and epidemiologic characteristics of *Cyclospora cayetanensis* infections during a 10-year period from patients consulting at the University Hospital, Honduras.

**Methods:**

Retrospective non interventional hospital-based study analyzed laboratory results from the period 2002 to 2011 of fresh and Ziehl-Nielsen carbolfuchsin stained routine stool samples received for parasitologic examination. Sporadically a sample with numerous oocysts was allowed to sporulate in 2.5 % potassium dichromate confirming the presence of bi-cystic bi-zoic oocysts.

**Results:**

A total of 35,157 fecal samples were examined during a ten-year span, of which a third (28.4 %) was stained by the Ziehl-Neelsen carbolfuchsin method diagnosing a total of 125 (1.3 %) *C.cayetanensis* infections. A statistically significant apparent seasonality was observed most years during May to August (range *p* < 0.036–0.001), with 83.3 % of 125 cases occurring in those rainy months. All *C. cayetanensis* cases came from urban poor neighborhoods; male/female relation was 1:1 except in 2006, when all patients were females (*p* = 0.05; r^2^ = 22,448). Forty four point eight percent of the stool samples were diarrheic or liquid and 65.6 % infections were identified in children 10 years old or less. Enteric helminths and protozoa co-infected *Cyclospora* positive patients in 52 instances.: 8 % *Ascaris lumbricoides*, 8 % *Giardia duodenalis*, 23.2 % *Blastocystis* spp. and less frequently *Entamoeba histolytica/E. dispar*, *Strongyloides stercoralis*, and *Trichuris trichiura*.

**Conclusions:**

Results suggest a **s**easonal pattern for *Cyclospora* infections diagnosed in a clinical setting during the rainy months in Tegucigalpa and surrounding areas. Community studies should be conducted to support or dispute these observations.

## Background

Cyclosporiasis is an enteric illness caused by an infection with *Cyclospora cayetanensis* oocysts, an intestinal apicomplexan protozoon endemic in many tropical and subtropical regions. It was initially reported in 1979 from Papua, New Guinea, and later recovered from immunocompetent travelers and ex-patriates in Nepal [1, 2]. It was classified to its actual taxonomic position in 1993 by demonstrating electron microscopy apicomplexan morphology and type of oocyst sporulation [3]. Several diarrheal outbreaks due to cyclosporiasis were initially published from the United States and Canada, identifying the probable source of infection in imported agricultural produce consumed uncooked such as raspberries, cilantro, basil leaves and in some cases by contaminated water [4, 5].

*Cyclospora cayetanensis* has now been identified worldwide as an intestinal pathogen in individuals of all ages. It is common in rural and urban areas of tropical and subtropical countries and has been implicated in sporadic outbreaks of gastrointestinal illness in developed countries [4, 5], in travelers to endemic countries [6, 7], in immunocompetent as well as in AIDS patients [8] and in children in endemic areas [9]. Subsequent studies have identified *C. cayetanensis* as an important cause of protracted enteric illness in humans in developed and developing countries [10]. However, in impoverished neighborhoods of some countries like Thailand and Haiti, high percentages of asymptomatic carriers with few oocysts in concentrated stools presume a degree of immune protection developed after early and continued exposure [11, 12].

The unsporulated oocysts are excreted in the feces of infected persons and require several days to more than a week in the environment under undefined conditions of temperature and humidity to sporulate and become infective [3]. The diagnosis consists of differentiation of *Cyclospora* oocysts from other intestinal apicomplexan parasites by different microscopic methods such as formalin-ethyl acetate concentration, Ziehl-Neelsen modified stain, fluorescence microscopy and laboratory sporulation [13].

It is worth mentioning the marked seasonality of its appearance in stool specimens, partially explained by ranges of temperature and humidity. In Guatemala, Nepal, Honduras, and Indonesia its frequency increases during the rainy season [2, 14, 15], in Perú and Haiti it appears during the cooler months of December to May and January to March, respectively [9, 12]. In Turkey, on the other hand, cases were clustered during 15 days in the warm months in summer [16].

The Parasitology Service of the public health University Hospital in Tegucigalpa, Honduras, serves as a diagnostic laboratory for all patients, both ambulatory and in-ward, who consult for medical attention and are requested to submit stools, blood and other pathological specimens (except biopsies) for parasitic infection identification. It functions as a parasitology research laboratory as well, for the School of Medical Sciences and different Residency programs that explore research projects on applied medical parasitology problems. It is staffed by one MD PhD parasitologist, one M.Sc. parasitologist, one microbiologist and three parasitology-trained and supervised laboratory technicians. After 1986 [14], when the first study on cryptosporidiasis identified this infection in children and patients diagnosed with the acquired immunodeficiency syndrome (AIDS) in Honduras, the Parasitology Service implemented the acid-fast modified carbolfuchsin staining method (AMS) to all stool samples of children less than 5 year old screening for *Cryptosporidium* spp. For adult patients and those with AIDS the method was applied on request by the attending physician and later (1990 to the present) to confirm *C. cayetanensi*s and/or *Cystoisospora belli* oocysts when such were observed by direct stool and stained smear examination of any patient. Results from studies conducted at the University Hospital in Tegucigalpa on the infection prevalence and epidemiology of intestinal apicomplexan parasites have been published locally [14, 17].

This study aimed to document results from a ten year (2002–2011) review of laboratory findings of *Cyclospora cayetanensis* infections as diagnosed during routine stool sample examination of patients who consulted at this tertiary care university hospital in Tegucigalpa.

The main purpose of the investigation was to document *C. cayetanensis *infections from Honduras, contribute with data on cyclosporiasis from this central american sub-region, and point out the distinct seasonality of its identification in the samples received and examined at the Parasitology Service. It is hoped that the information obtained will promote interest to investigate further on its epidemiology, distribution and clinical characteristics in this sub-region of Latin America.

### Ethical approval

Ethical and scientific approval of the protocol was obtained from the Ethics Committee of the School of Medical Sciences of the National Autonomous University of Honduras, as well as by the Pediatric Department of the University Hospital, both in the capital city, Tegucigalpa. It was designed as a descriptive and non interventional study in nature, where retrospective data were obtained anonymously from the laboratory yearly registry book. Therefore, the Ethics Committee considered this study observational, in a university hospital setting, and although patient permission was not sought beforehand, the gathering and analyses of data were of no threat to patients; the information generated would have a scientific value and the proposal was approved in the understanding that all information retrieved would be kept confidential.

## Methods

### Study site description

The University Hospital (UH) is a tertiary level care public facility in the capital city of Tegucigalpa, Honduras associated to the School of Medical Sciences of the National Autonomous University (UNAH). It has a 1055 bed capacity and different specialty outpatient clinics attending individuals of all ages, of low socio-economic strata from the capital city, neighboring areas and referrals from anywhere in the country. The Parasitology Service (PS) at the Department of Clinical Laboratory attends outpatients as well as hospitalized patients five days a week all year round from 7:00 a.m. to 2:00 p.m. (shift A), which is the time frame reported here. It receives and examines an average of 4000 fecal samples a year. Stool results from two other shifts were not included in this study. The laboratory is equipped to provide diagnostic services based on microscopical observations by different approved routine methods. Stool specimens are received fresh, in appropriate glass containers labeled with patient’s identification and accompanied by a printed request form registering date, name, age, sex of the patient and attending clinic or hospital ward and a space to annotate laboratory results.

### Laboratory stool examination protocols

Fecal specimens, predominantly one sample per patient, are received at the PS during shift A as part of the daily routine examination requests to both out- and inpatient population consulting at the UH for medical care. Following the PS protocols in place, fresh stool samples are examined within the first three hours of reception. Routine methodology consists of a macroscopic examination to register stool consistency, presence of blood, mucus, or adult worms or segments. A wet mount in physiologic saline solution as well as a wet mount in Lugol’s iodine are routinely examined microscopically and any parasitic infection registered. Fecal samples belonging to children five year old or less, as well as requests by clinicians are routinely stained with AMS, a protocol implemented at the PS since 1990 mainly to confirm *Cryptosporidium* spp. and *Cystoisospora belli* infections in children and adults [18]. Stools from referral patients at the HIV/AIDS unit are also stained with AMS as are those samples where *Cyclospora* oocysts are suspected in a direct saline smear. On average a third of all monthly fecal samples received routinely are prepared as thin smears, air dried, fixed and stained with AMS. The stained smears are searched microscopically under high dry magnification (400x) and any suspected oocysts of either human intestinal apicomplexa (*Cryptosporidium* spp*.*, *C. belli* and *C. cayetanensis*) are identified under oil immersion (1000x). *Cyclospora* oocysts are differentiated by size, between 8 and 10 μm in diameter and by staining characteristics.

Sporadically a few positive stool samples with numerous *C. cayetanensis* and/or *C. belli* oocysts have been sporulated in the laboratory to confirm species identification and for student demonstration. Briefly, a part of the positive stool sample was placed in 2.5 % potassium dichromate solution, thoroughly suspended by mixing with a wooden applicator and transferred to a large covered Petri dish kept at room temperature. At intervals a convenient stool suspension amount was transfered between slide and coverslip and examined under 40X to identify sporulated *Cyclospora/C. belli* oocysts. *Cystoisospora belli* oocysts sporulated readily in 1 or 2 days; *C. cayetanensis* oocysts took longer (time was not registered given that the main purpose was to obtain oocyst sporulation), but sporulated oocysts observed microscopically under oil immersion contained two sporocysts with two sporozoites each [1, 3].

### Retrospective parasitology data

For the retrospective *C. cayetanensis* analysis we reviewed the daily Registry Book of the PS for the years 2002–2011, retrieving data such as total monthly and yearly stool examinations and number of samples stained by AMS. All enterings of positive results for the presence of *C. cayetanensis* oocysts as confirmed by the acid-fast modified stain were highlighted and annotated in a predesigned questionnaire tool including the following information: year and month, patient file number, age, sex, stool consistency, and parasitology results. No patient name was registered; their respective file numbers were written down only to avoid same patient registry results in those occasional instances of serial stool examination.

### Data on climatic changes

Ten-year (2002–2011) average precipitation and temperature data were kindly requested and were made available from the National Meteorology Observatory Station Number 78720 (1000 m altitude). According to monthly average precipitation and temperature data from the capital city, the rainy season started on average at the end of April, going from 7.42 mm^3^ precipitation in March to two peaks in June (190.57 mm^3^) and September (140.94 mm^3^) respectively, dropping abruptly to 10.5 mm^3^ towards December. Temperature in June was 23.1 °C, with a yearly average of 22.3 °C.

### Statistical analysis

Data on results of *C. cayetanensis* oocyst identification from the retrospective study such as month and year, age, sex, stool consistency and other intestinal parasites diagnosed were entered and analysed by the program SPSS versión 21® (IBM, Armonk, New York USA, 2012) and Graphpad Prism versión 5.0c (La Jolla, CA., 2010). Percentages were calculated for age, sex, number of slides stained by acid fast carbolfuchsin in relation to total stool samples received monthly and yearly. Association between variables was evaluated using a test of independence by Chi-square test (*x*2). Linear regression was used for determining potential associations between climate and *Cyclospora* cases. A *p* value of <0.05 was considered significant.

## Results

As an example of consultation volume, total patient attentions (excepting maternal deliveries) for the year 2010 at the University Hospital amounted to 152,078; 97,789 were outpatient adult consultations of which 14,324 were hospitalized; the pediatric consultations totaled 54,289 of which 15,622 were hospitalized. From that total, only 3649 (2.4 %) patients were requested to submit a stool sample in that particular year(shift A). Similar hospital data from previous years were not available.

### Retrospective observations

**Fig. 1:**
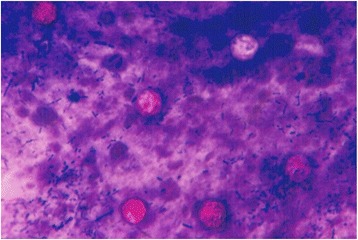
*Cyclospora cayetanensis* oocysts from fecal smear stained by the acid fast modified carbolfuchsin method. Oocysts were identified by size, between 8 to 10 μm, morphology and irregular staining characteristics. X 1000

Oocysts of *C. cayetanensis* when present in the stools were usually few in number, microscopically identified as round, refringent organisms showing a well delimiting oocyst wall, with regular granulated contents, measuring 8–10 μm in diameter. In the AMS the wall of the oocyst had some times a wrinkled appearance, with differences in staining intensity, some slight pink, or deep purple and some unstained, all characteristics used to narrow the recognition as *Cyclospora* oocysts (Fig. 1).

**Fig. 2:**
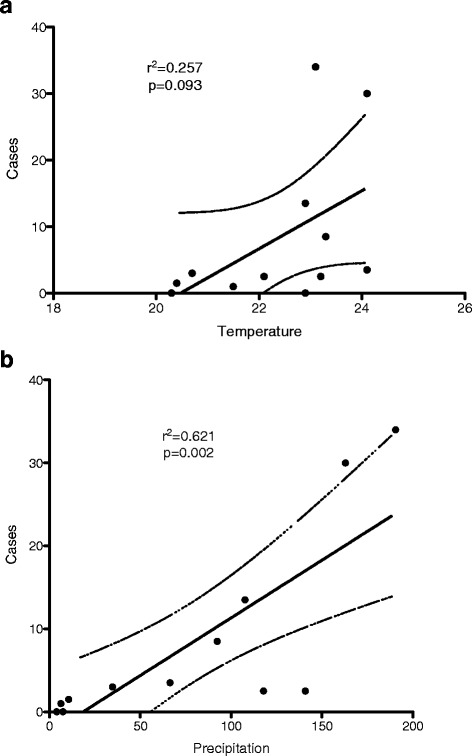
Linear regression of temperature (**a**), precipitation (**b**) and *Cyclospora cayetanensis* hospital cases, 10-year observations

A yearly summary of results for the retrospective data of total routine fecal examinations and total AMS stains performed at the PS during shift A between the years 2002 and 2011 are presented in Table 1. A total of 35,157 stool specimens were examined with an average of 3515 samples per year. Total AMS examined during same time span was 10,004 (28.4 %), slightly less than a third of all fecal samples received. One hundred and twenty five (1.3 %) *Cyclospora* infections were identified by AMS between 2002 and 2011; at yearly level *Cyclospora* case incidence presented a cyclical pattern with a slight variation in frequency by month and by year, with a pick in the higher precipitation months of May and June, slowly dropping in July and August, so that 104 of 125 (83.3 %) cases occurred in the 10- year span in those four months. A statistical difference was found in all years except 2002, 2006 and 2008 for the months of May (2005, *p* = 0.007; and 2007, *p* = 0.001), June (2003, *p* = 0.010; 2004, *p* = 0.025; 2005, *p* = 0.007; 2010, *p* = 0.006; 2011, *p* = 0.001)) and July (2009, *p* = 0.036) (Table 1). Oocysts started to be apparent in the stools around the end of April in two occasions, one case each in 2008 and 2011, respectively, or beginning of May, reaching a peak later in May (23 cases, 18.4 %), and June (47 cases, 37.6 %) to gradually diminish in July (20 cases, 16 %) and August (14, 11.2 %), with sporadic cases (22, 17.6 %) from September to February, none in January or March and seldom in April (Table 1). From 2002 to 2011 there were an average of 12.5 cases per year (range 4.8–16). There was an association between precipitation (r^2^ = 0.621; F = 16.384; *p* = 0.002) but not temperature, and incidence of *C. cayetanensis* as analyzed by linear regression and shown in Fig. 2a and b. The increase in the number of cases would be proportional to the amount of precipitation, that is to say, with each 1 % precipitation the number of cases would increase proportionally 0.139 cases (Fig. 2b); the level of confidence for such prediction would be 62.1 %, (r^2^ = 0.621). On the other hand, temperature did not show such an association (Fig. 2a) (r^2^ = 0.257; F = 3.452; *p* = 0.093).

**Table 1:** Totals and percentages of stool exams, total acid fast stained samples and *Cyclospora* cases by month and year during 10-year retrospective review of laboratory data, Honduras

Year	Total/AMS	My	Jn	Jl	Aug	Sp	Oc	Nov	*P*-value
2002	4392/894 (20.3)	0	2	1	0	0	1	1	NS
2003*	3848/1047 (27.2)	1	6*	3	0	1	0	0	0.010
2004*	4310/1226 (28.4)	2	7*	2	0	1	0	2	0.025
2005*	2878/1226 (42.5)	5*	6*	0	0	0	0	2	0.007
2006	3345/864 (25.8)	1	2	2	3	2	0	0	NS
2007*	3205/852 (26.5)	7*	1	1	0	1	1	1	0.001
2008	2738/763 (27.8)	2	3	1	0	0	0	0	NS
2009*	2789/904 (32.4)	2	1	6*	3	0	0	0	0.036
2010*	3649/1024 (28.8)	1	7*	2	5	0	2	0	0.006
2011*	4003/1204 (30.0)	2	12*	2	3	0	0	0	0.001
Total^a^	35157/10004 (28.4)	23	47	20	14	5	4	6	

^a^one case in February 2002, no cases in January and March, 1 case each April 2008 and 2011, and 1 case each December 2003, 2004 and 2006, respectively

*Statistical significant association between months and years for the presence of *Cyclospora* oocysts, established by Chi-square test (*p* < 0.05)

*NS* not significant

In 3 (2.4 %) out of 125 *Cyclospora* cases age was not provided, 60/122 (49.1 %) positive samples were from children 0 to 5 year-old and 80/122 samples (65.6 %) with *C. cayetanensis* oocysts came from children 0 to 10 year-old (Table 2). The male/female risk ratio during 10 years was 1:1, except for the year 2006 when all eleven cases were found in female patients (*p* = 0.05; *x* ^2^ = 22,448) (Table 3). There was no variation in patient characteristics, like grouping by sex nor clustering in a different neighborhood or living far outside the surrounding area of Tegucigalpa; however one limitation was the unavailability of data to compare different age groups statistically. Not all stool samples positive for *Cyclospora* were graded as diarrheic or liquid, 69 (55.2 %) were formed or soft (Table 4). In a total of 12 instances the presence in stools of moderate or abundant mucus was registered, as were cases with occult blood (one stool sample) and macroscopic blood (one stool sample). No segments or adult worms were recovered from any of the 125 *Cyclospora* positive stools. The number of diarrheic or liquid stools examined with no oocysts recognition was not investigated. In 56/125 fecal samples (43.7 %) only *Cyclospora* oocysts were identified, usually few in number but numerous occasionally; no estimation of infection intensity by counting oocysts in the stained smear was performed. In 15/125 (12 %) samples leucocytes were also present, Charcot-Leyden crystals in 10 (8 %) and in 52 instances one or more co-infections with different intestinal parasite species and commensals were recognized (Table 2). Seven out of 10 *Ascaris lumbricoides* infections were in children less than 10 years old, 5 of which were heavy with 100 eggs or more in 2 mg of feces, followed by *G. duodenalis* (7 out of 10 infections), one *Strongyloides stercoralis* infection, one *Balantidium coli* and one severe (800 eggs in a 2 mg fecal smear) *Trichuris trichiura* infection; *Hymenolepis nana* eggs were identified in a 39 y old patient (not shown in Table). The most common co-infecting organism was *Blastocystis* spp*.* (29 instances, 23.2 %); cysts of commensal protozoa were identified as *Entamoeba coli* in 15 fecal samples, *Entamoeba hartmanni* in 2 cases, *Endolimax nana* in 4 samples, *Chilomastix mesnili* in 3 samples, *Entamoeba histolytica/E. dispar* in one and *Trichomonas hominis* trophozoites in 4 (not shown in Table).

**Table 2:** Distribution of *Cyclospora cayetanensis* by age strata and concurrent parasitic infections, 2002–2011

Age strata	*Cyclospora *cases (%)	Leu	Mixed parasitic infections by age strata					
			A.l.	S.s.	G.d.	B.spp.	C.b.	Ch.L.
0–11 m	5 (4.0)	2	0	0	0	0	0	0
12–23 m	8 (6.4)	2	0	0	0	0	0	1
24–35 m	16 (12.8)	2	1	0	2	0	0	1
3–5 y	31 (24.8)	2	4	1	3	8	0	1
6–10 y	20 (16.0)	2	2	0	2	8	0	2
11–20 y	13 (10.4)	1	1	1	1	7	0	1
21–35 y	15 (12.0)	2	0	0	1	4	1	2
36–49 y	5 (4.0)	1	1	0	0	1	0	1
≥50 y	9 (7.2)	0	0	0	0	1	0	0
No age recorded	3 (2.4)	1	1	0	1	0	0	1
Totals (%)	125	15 (12.0)	10 (8.0)	2 (1.6)	10 (8.0)	29 (23.2)	1 (0.8)	10 (8.0)

*Abbreviations*: *Leu* leukocytes, *Al Ascaris lumbricoides*, *Ss Strongyloides stercoralis*, *Gd Giardia duodenalis*, *B spp. Blastocystis* spp., *Cb Cystoisospora belli*, *ChL* Charcot-Leyden crystals, *m* months, *y* years

**Table 3:** Distribution of *Cyclospora* oocysts by patient sex, 10-year period

Year	Male	Female	Total *C. cayetanensis* cases
2002	4 (6.5)	2 (3.1)	6 (4.8)
2003	5 (8.2)	7 (11.1)	12 (9.6)
2004	6 (9.8)	9 (14.3)	15 (12.0)
2005	9 (14.7)	4 (6.3)	13 (10.4)
2006	0	11* (5.4)	11 (8.8)
2007	6 (9.8)	6 (9.5)	12 (9.6)
2008	3 (4.9)	4 (6.3)	7 (5.6)
2009	9 (14.7)	4 (6.3)	13 (10.4)
2010	10 (16.4)	6 (9.5)	16 (12.8)
2011	9 (14.7)	10 (15.9)^a^	20 (16.0)
Totals	61 (48.8)	63 (50.4)	125

*Significant association established by Chi-square test (*p* < 0.05; *x* ^2^ = 22,448)

all female cases only in 2006

^a^Sex not registered in one case

**Table 4:** Stool consistency, presence of mucus and blood (macro- and microscopic) in 125 positive *Cyclospora* cases, Honduras

Stool consistency	Year of observation 2002–2011										Totals (%)
	02	03	04	05	06	07	08	09	10	11	
Formed	2	3	3	7	6	5	1	3	2	5	37 (29.6)
Soft	0	3	6	5	2	4	4	4	1	3	32 (25.6)
Diarrheic	0	5	4	1	1	2	1	1	5	7	27 (21.5)
Liquid	4	1	2	0	2	1	1	5	5	1	22 (17.5)
Mucus	0	1	2	0	0	0	2	3	0	4	12 (9.5)
^a^Occult blood/Blood macro	0	0	1	1	0	0	0	0	0	0	2 (1.5)

^a^As requested by physician, not performed routinely

According to a summary monthly average precipitation and temperature data from the capital city provided by the National Meteorology Observatory Station 78720 (1000 m elevation), the rainy season started on average at the end of April, going from 7.42 mm^3^ precipitation in March to two peaks in June (190.57 mm^3^) and September (140.94 mm^3^) respectively, dropping abruptly to 10.5 mm^3^ towards December. Temperature for the 10-year observation period showed slight variation, with a low median in January of 20.3 °C to a maximum median in April of 24.1 ° C. Median temperatures for June, July, August and September were 23.1 °C, 22.9 °C, 23.3 °C and 23.2 °C, respectively. Linear regression of univariant analysis showed a significant association between precipitation and the incidence of *C. cayetanensis* findings (Fig. 2b: r^2^ = 0.621; F = 16.384; *p* = 0.002); such association was not found when comparing average temperature and *Cyclospora* cases (Fig. 2a: r^2^ = 0.257; *p* = 0.093).

## Discussion

This study describes the occurrence of *C. cayetanensis* infection diagnosed in individuals of all ages attending a tertiary care hospital in the capital city of Honduras from 2002 to 2011. Because the report is based on *Cyclospora* oocyst finding during routine examination of stool samples, some underestimation in the frequency data is possible since only one stool specimen per patient was examined, about a third of the samples received monthly were stained by the AMS as explained previously, results from other two laboratory shifts are not included and no concentration techniques that might have identified light infections were applied. Although the methods used are the ones implemented for the daily routine at the PS and all samples were examined with similar thoroughness thus assuring uniformity of performance, some infections could have been missed. No other public health laboratory from any region in the country has any documented data of *C. cayetanensis* infection probably because of lack of awareness and familiarity with the parasite; only one limited study in the community showed 2.7 % (4/144) of diarrheic children infected with *C. cayetanensis* in a marginal barrio of the capital city [19].

One goal of this study was to summarize the current status of *C. cayetanensis* infection diagnosis as has been observed in Honduras during a 10- year span from 2002 to 2011. A second important objective was to demonstrate a significant relationship of *Cyclospora* oocysts presence in stools during the months of higher precipitation for the years 2002–2011, which was, however, not associated with temperature. Data of the first 12 years (1990–2001) of hospital case observations were published locally and it was apparent then that the infection had a consistent seasonal pattern of occurrence (84.6 % of 96 cases) during the months of May to August, which coincide with the beginning of the rainy season [14], a pattern persistent in the present study. In other words, for 22 consecutive years the recognition of *C. cayetanensis* oocysts has been somehow associated with an increase in precipitation. This intriguing fact is consistent with reported cyclosporiasis seasonality in other countries such as Guatemala, Nepal, Indonesia and Jordan [2, 15, 20], while in Perú and Haiti [9, 12] the infection was found to occur in cooler months. One report from Turkey [16] seemed exceptional since the infection was observed during 15 days of a warm and dry month that year, the mode or source of contamination in that apparent outbreak was not investigated. Certain environmental factors in endemic developing countries as yet to be identified such as ranges in humidity and temperature most likely facilitate sporulation and oocyst survival as long as those factors remain. Rainfall can spread the pathogen from a soil contaminated environment with human feces [1] to water reservoirs or contaminate water systems [2, 21], or irrigated produce consumed raw [22, 23] facilitating pathogen transmission and reaching susceptible hosts such as unaware travelers, children, non immune or immunodeficient individuals who consequently become infected. The quality of water is also important as it relates to diarrheal disease. In Honduras only 34 % of the sanitary facilities are connected to a sewer, of which 62 % are in urban areas leaving rural areas and marginal neighborhoods with higher soil and water pollution [24]. Piped untreated water for human use is distributed either through direct reticulation inside (41 %) or by provision through public taps outside (45 %) the house, and is available to 86 % of the homes in Honduras [24]. In the past it was found that households in poor communities with children 5 year-old or less that bought and stored water from distribution trucks were 2.5 times more likely to have diarrhea than children from households with water in the home [25].

With this study we document up to date data on cyclosporiasis as observed in this Central American country. From the seven countries that constitute Central America (geographically, not political) only Guatemala with several reports on cyclosporiasis as relate to exported produce and hospital and health center cases [21, 26] and Costa Rica [23, 27, 28] had publications easy retrievable for perusal. In developing countries such as ours greater awareness of the parasite, its biology, geographical distribution and factors that facilitate its different modes of transmission and the disease it causes are research subjects of important public health interest, and should be therefore of easy access in the published literature, so as to take measures to protect populations at risk, prevent parasite spreading and contamination and facilitate implementation of effective parasite surveillance.

This study did not focus on clinical aspects of cyclosporiasis, although about half the cases consulting at the hospital emergency ward complained of gastrointestinal symptoms and presented with diarrheic or liquid stools. The distribution of *Cyclospora* cases that were seen at the emergency ward tended to be more common in children in the 2 to 10-year-old group (63.6 %) from marginal barrios and surrounding rural areas suggesting an early contamination with oocysts but who were in some cases co-parasitized by agents known to cause gastroenteric symptoms. Moreover, no other etiological agents of diarrhea were investigated, thus making it not possible to affirm that *Cyclospora* was the cause of the gastroenteric illness. The number of infected hosts in a given neighborhood or community plus the contamination by infected individual with pathogen shedding along with the presence of susceptible hosts may all contribute to the transmission of the pathogen which characterizes the dynamics of infectious diseases. Studies conducted in health centers or clinics elsewhere have found cyclosporiasis more common in children five years old or less [3, 9, 29]; still reasons why this happened are not clear and warrant further comprehensive research.

Intestinal parasites are very common among different populations in Honduras even though published evidence is sparse, with limited methodology and few conducted in the community [19, 30–33]. Age-infection profiles identifies children at greater risk of infection with multiple parasite species. From 266 children 6 year-old or less with and without diarrhea from a marginal barrio and two rural communities, 60.9 % had *G. duodenalis* cysts, 48.8 % were infected with *A. lumbricoides*, *Cryptosporidium* spp. oocysts were present in 61 % of 18 children less than a year old and 124 children were co-infected with 5–10 different species of helminths and cysts of commensal protozoa [30]. Most intense infections with *A. lumbricoides* and *T. trichiura* was found in children 2 to 12 year-old living in four rural communities, where the overall prevalence was 45 % (95 % CI 39.0–51.9) and 38 % (95 % CI 31.8–44.4) for ascariasis and trichuriasis, respectively [31]. Among 133 adult participants asymptomatic but infected with human immunodeficiency virus living in five cities of Honduras, the overall prevalence of intestinal parasites ranged from 58.6 % in Tegucigalpa to 90 % in Tela/La Ceiba (95 % CI = 59.2–82.9) [32]. Undernutrition and anemia were the underlying most important characteristics among 13 hospitalized children 12 year-old or less suffering from a severe trichuriasis syndrome and multiparasitism [33]. In an effort to place neglected infectious diseases high in the Ministries agendas from countries in Latin America and the Caribbean, the Panamerican Health Organization (PAHO) is enhancing its attention to five countries including Honduras, considered a key country where rural communities and vulnerable populations are infected with some of the 14 listed neglected diseases identified by the World Health Organization, raising awareness of their overall negative impact and focusing on implementing community-participation, multi-disease, inter-programmatic approaches, having as goal to reduce the burden of disease to improve health status and quality of life in vulnerable populations [34].

Data derived from the approach we chose for the study are necessarily limited. All of the samples of this study came from hospital patients, a fact that may have introduced a selection bias and consequently may not reflect the true prevalence of *C. cayetanensis* in Honduras. However, some critical information may have been provided on *Cyclospora* epidemiology characteristics in the setting of a public health care hospital and among economically poor population in Tegucigalpa. Important gaps still exist; as mentioned in a recent publication, such unawareness of this parasitic infection can lead to misdiagnosis and incorrect treatment, the administration of antibiotics or self medication only exacerbating the problem [35]. Moreover, extra intestinal complications have been documented associated with *Cyclospora* infections such as Guillian-Barre syndrome [36] biliary disease [37], patients infected with human immunodeficiency virus [8] and reactive arthritis syndrome [38]. Delayed diagnosis, inadequate knowledge among health personnel and lack of proper treatment and follow-up affect children who are the most frequent sufferers. Steps should be taken to eliminate barriers by increased education of health professionals in parasitic diseases, conduct rigorous studies in the community, and unify a workable consistent reporting protocol for the study of clinical cases.

## Conclusions

In summary, the present study confirms the seasonality of cyclosporiasis as seen in patients consulting at a hospital in Tegucigalpa, Honduras. Oocysts of C. cayetanensis are difficult to diagnose and a modified carbolfuchsin acid fast method should be performed in stools in suspected cases. Well planned and carefully executed future community studies should clarify aspects on the epidemiology of this apicomplexan parasite as in its role in diarrheal disease. Better awareness of this and other parasitic infections among health personnel is of urgent need.

## References

[1] Chacín-Bonilla L (2010). Epidemiology of *Cyclospora cayetanensis*: a review focusing in endemic areas. Acta Tropica.

[2] Shields JM, Olson BH (2003). *Cyclospora cayetanensis*: a review of an emerging parasitic coccidian. Internat J Parasitol.

[3] Ortega YR, Sterling CR, Gilman RH (1998). Cyclospora cayetanensis. Adv Parasitol.

[4] Herwaldt BL, Ackers ML (1997). An outbreak in 1996 of cyclosporiasis associated with imported raspberries. N Engl J Med.

[5] Herwaldt BL (2000). *Cyclospora cayetanensis*: A review, focusing on the outbreaks of cyclosporiasis in the 1990s. Clin Infect Dis.

[6] Ramírez-Olivencia G, Herrero MD, Subirats M, Rivas González P, Puente S (2008). *Cyclospora cayetanensis* outbreak in travelers to Cuba. Enferm Infecc Microbiol Clin.

[7] Kansouzidou A, Charitidou C, TVarnis T, Vavatsi N, Kamaria F (2004). *Cyclospora cayetanensis* in a patient with travelers’ diarrhea: case report and review. J Travel Med.

[8] Tsang OT, Wing-cheuk Wong R, Hoi-shiu Lam B, Man-chun Chan J, Tsang K, Leung W (2013). *Cyclospora* infection in a young woman with human immunodeficiency virus in Hong Kong: a case report. BMC Res Notes.

[9] Bern C, Ortega Y, Checkley W, Roberts JM, Lescano AG, Cabrera L (2002). Epidemiologic differences between cyclosporiasis and cryptosporidiosis in Peruvian children. Emerg Inf Dis.

[10] Karanja RM, Gatei W, Wamae N (2007). Cyclosporiasis: an emerging public health concern around the world and in Africa. African Hlth Sci.

[11] Thima K, Mori H, Praevanit R, Mongkhonmu S, Waikagul J, Watthanakulpanich D (2014). Recovery of *Cyclospora cayetanensis* among asymptomatic rural Thai schoolchildren. Asian Pacific J Trop Med.

[12] Eberhardt M, Nace EK, Freeman AR, Streit TG, Da Silva A, Lammie P (1999). *Cyclospora cayetanensis* infections in Haiti: a common occurrence in the absence of watery diarrhea. Am J Tropical Med Hyg.

[13] Eberhard ML, Pieniazek NJ, Arrowood MJ (1997). Laboratory diagnosis of *Cyclospora* infections. Arch Pathol Lab Med.

[14] Kaminsky RG (2002). Epidemiological comparison of intestinal apicomplexan infections at the University Hospital, Honduras. Rev Méd Hondur.

[15] Fryauff DJ, Krippner R, Prodjodipuro P, Ewald C, Kawengian S, Pegelow K (1999). *Cyclospora cayetanensis* among expatriate and indigenous populations of West Java, Indonesia. Emerg Inf Dis.

[16] Ozdamar M, Hakk E, Turkoglu S (2010). Higheoccurrence of cyclosporiasis in Istanbul, Turkey, during a dry and warm summer. Parasit Vectors.

[17] Kaminsky RG (1997). *Cyclospora cayetanensis*: new intestinal apicomplexan parasite, update and cases presentation, University Hospital. Rev Méd Hondur.

[18] Kaminsky R. Parasitology Manual. Techniques for Primary Health Care laboratories and for the diagnosis of Neglected Infectious Diseases. 3rd ed. Panamerican Health Organization, Institute for Infectious Diseases and Parasitology Antonio Vidal Honduras; 2014.

[19] Arima Y, Kaminsky RG, Ávila G, Casiano-Colón A, Guthrie BL, DiGiacomo RF, Jacobs J (2011). New and old agents associated to diarrhea in children in Honduras. Rev Méd Hondur.

[20] Nimri LF (2003). *Cyclospora cayetanensis* and other intestinal parasites associated with diarrhea in a rural area of Jordan. Int Microbiol.

[21] Bern C, Hernandez B, Lopez MB, Arrowood MJ, de Mejía A, Merida AM (1999). Epidemiologic studies of *Cyclospora cayetanensis* in Guatemala. Emerg. Infect. Dis.

[22] Doller PC, Dietrich K, Filipp N, Brockmann S, Dreweck C, Vonthein R (2002). Cyclosporiasis outbreak in Germany associated with the consumption of salad. Emerg Infect Dis.

[23] Calvo M, Carazo M, Arias ML, Chaves C, Monges R, Chinchilla M (2004). Prevalence of *Cyclospora* sp., *Cryptosporidium* sp., microsporidia and determination of fecal coliforms in fresh fruits and vegetables consumed raw in Costa Rica. Arch Latinoam Nutr.

[24] World Health Organization (2013). Country Cooperation Strategy.

[25] Figueroa M, Poujol E, Cosenza H, Kaminsky R (1990). Etiology of diarrea in children from three honduran communities. Rev Méd Hondur.

[26] Pradesaba RA, González M, Piedrasanta E, Mérida C, Contreras K, Vela C (2001). *Cyclospora cayetanensis* in three populations at risk in Guatemala. J Clin Microbiol.

[27] Cedeño TC (2002). *Cyclospora cayetanensis*: description of the first case in the Hospital San Rafael de Alajuela. Acta Med Costaricense.

[28] Chinchilla MC, Guerrero Bermúdez OM, Reyes Lizano L, Castro Castillo A (1999). *Cyclospora cayetanensis*: revisión and presentation of first human case in Costa Rica. Acta Méd Costaricense.

[29] Madico G, McDonald J, Gilman RH, Cabrera L, Sterling CR (1997). Epidemiology and treatment of *Cyclospora cayetanensis* infection in Peruvian children. Clin Infect Dis.

[30] Kaminsky RG (1991). Parasitism and diarrhea in children from two rural communities and a marginal barrio, Honduras. Trans R Soc Trop Med Hyg.

[31] Smith H, Kaminsky RG, Niwas S, Soto RJ, Jolly P (2001). Prevalence and intensity of infections of *Ascaris lumbricoides* and *Trichuris trichiura* and associated socio-demographic variables in four Honduran communities. Mem Inst Oswaldo Cruz, Rio de Janeiro.

[32] Kaminsky RG, Soto RJ, Campa A, Baum M (2000). Intestinal parasitic infections and eosinophilia in an human immunodeficiency virus positive population in Honduras. Mem Inst Oswaldo Cruz Rio de Janeiro.

[33] Kaminsky RG, Valenzuela R, Ábrego FC (2015). Growth retardation and severe anemia in children with *Trichuris* dysenteric syndrome. Asian Pacific J Trop Biomed.

[34] Ault SK (2007). Pan American Health Organization’s Regional Strategic Framework for addressing neglected diseases in neglected populations in Latin America and the Caribbean. Mem Inst Oswaldo Cruz, Rio de Janeiro.

[35] Sánchez-Vega JT, Cabrera-Fuentes HA, Romero-Olmedo AJ, Ortiz-Frías JL, Sokolina F, Barreto G (2014). Case Report: *Cyclospora cayetanensis*: This emerging protozoan pathogen in Mexico. Am J Trop Med Hyg.

[36] Richardson RF, Remler BF, Katirji B, Murad MH (1998). Guillian-Barre syndrome after *Cyclospora* infection. Muscle Nerve.

[37] Sifuentes-Osornio J, Porras-Cortes G, Bendall RP, Morales-Villarreal F, Reyes-Teran G, Ruiz-Palacios GM (1995). *Cyclospora cayetanensis* infection in patients with and without AIDS: biliary disease as another clinical manifestation. Clin Infect Dis.

[38] Connor B, Johnson E, Soave R (2001). Reiter syndrome following protracted symptoms of *Cyclospora* infection. Emerg Inf Dis.

